# Novel Hawai’i and Pacific Island circulating clusters of *Mycobacterium intracellulare* subsp. *chimaera*

**DOI:** 10.1128/aem.00269-26

**Published:** 2026-06-10

**Authors:** Rachel N. Wilsey, Liang-Hao Ding, Stephanie N. Dawrs, Chelsea K. Raulerson, Grant J. Norton, Ravleen Virdi, Nabeeh A. Hasan, L. Elaine Epperson, Stephen T. Nelson, Brady Holst, Yvonne L. Chan, Kara Kitamura, Jonathan D. Awaya, Sally V. Irwin, Steven Cornell, Mark B. Cannon, Sara Anglin, Marisa K. Chelius, Robert Hutchinson, Sarah Kern, Abi Stearns, Michael Strong, Edward D. Chan, James L. Crooks, Jennifer R. Honda

**Affiliations:** 1 Department of Cellular and Molecular Biology, School of Medicine, University of Texas Health Center at Tyler 12341 , Tyler, Texas, USA; 2 Center for Genes, Environment, and Health, National Jewish Health 2930, Denver, Colorado, USA; 3 Department of Geology and Geophysics, University of Utah 7060, Salt Lake City, Utah, USA; 4 Division of Biostatistics and Bioinformatics, National Jewish Health 2930, Denver, Colorado, USA; 5 ‘Iolani School 146116, Honolulu, Hawaii, USA; 6 Kapa’a High School, Kapa’a, Hawaii, USA; 7 Biology Department, University of Hawai’i at Hilo, Hilo, Hawai, USA; 8 Department of Science, Technology, Engineering, and Mathematics, University of Hawai’i Maui College, Kahului, Hawaii, USA; 9 Seabury Hall 255579 , Makawao, Hawaii, USA; 10 Faculty of Sciences, Brigham Young University 14678, Laie, Hawaii, USA; 11 Kailua High School 258929 , Kailua, Hawaii, USA; 12 Island School 810940 , Lihue, Hawaii, USA; 13 Kamehameha Schools 88326, Honolulu, Hawaii, USA; 14 Wai’anae High School, Wai’anae, Hawaii, USA; 15 Stearns Family Homeschool, Lihue, Hawaii, USA; 16 Division of Pulmonary Sciences and Critical Care Medicine, University of Colorado Anschutz Medical Campus 129263, Aurora, Colorado, USA; 17 Rocky Mountain Regional Veterans Affairs Medical Center 19982 , Aurora, Colorado, USA; 18 Academic Affairs, National Jewish Health 2930, Denver, Colorado, USA; Georgia Institute of Technology, Atlanta, Georgia, USA

**Keywords:** nontuberculous mycobacteria, *Mycobacterium intracellulare *subsp.*chimaera*, environment, whole genome sequencing, Hawai’i

## Abstract

Nontuberculous mycobacteria (NTM) are environmental microorganisms for which large, systematic studies of niche diversity are lacking. We performed a semi-longitudinal state-wide sampling campaign (2015–2019) for environmental NTM across Hawai’i. A volunteer network collected 2,334 water biofilms, soil, and dust samples from built (*n* = 1,946) and natural (*n* = 388) sites. Of these, 541 contained culturable NTM (23%) and per island hotspots identified. Of 74 NTM species recovered, the most prevalent rapid growing mycobacteria (RGM) were *Mycobacterium porcinum*, *Mycobacterium chelonae,* and *Mycobacterium abscessus. Mycobacterium intracellulare* subsp. *chimaera* was the most frequently isolated slow growing mycobacteria (SGM). Our longitudinal analyses indicate widespread colonization of diverse niches by species less associated with lung infections such as *M. chelonae*. In contrast, household water biofilms tended to be reliable niches for *M. abscessus* and *M. avium* complex species colonization across the 5-year study. Analysis of 590 deidentified lung samples from Hawai’i and other Pacific Islands revealed *M. chimaera* as the most frequently isolated species (40%, 238/590). Phylogenetic analysis of environmental and lung *M. abscessus* suggests most cluster within dominant circulating clone 1 (DCC1). Contrastingly*,* most Hawai’i and other Pacific Island *M. chimaera* were distinct from previously studied European isolates, leading to the identification of two novel clusters of phylogenetically related strains we have termed Pacific Island Circulating Cluster 1 and 2 (PCC1, PCC2). PCC1 consists exclusively of Hawai’i/Pacific Island isolates, while PCC2 was enriched by lung samples and was mostly collected from Hawai’i/Pacific Islands. These data reveal the genetic diversity, ecological niches, and potential reservoirs of NTM in varied Hawai’i ecosystems.

**IMPORTANCE:**

Nearly one in four environmental Hawai’i samples tested positive for any NTM species, with hotspots often overlapping population centers. Recovery of any NTM species occurred just as often from natural settings as homes and public buildings, highlighting exposures as a normal part of life. Soil was the most common reservoir for NTM colonization, but we distinguish NTM species pertinent to lung disease that were far more likely to be found in water biofilms, such as showerheads and kitchen sinks. No single species dominated the environment; yet, the type of NTM found in water systems closely mirrored those recovered from patients’ lung samples. Genetic analyses revealed that Hawai’i harbors distinct, locally circulating strains, including lineages not linked to known hospital outbreaks. Together, these findings improve our understanding of where precarious exposures can occur and inform public health strategies to reduce exposures by highlighting niches that are common hotspots for NTM colonization.

## INTRODUCTION

Interest in the interactions between nontuberculous mycobacteria (NTM) and the natural environments in which they are found began over 70 years ago (1, 2) and has continued to gain traction. In particular, we now know NTM isolated from lung samples differ geographically (3), and thus, NTM responsible for lung infections should be similar to those represented in soil and water within individual environmental niches. However, demonstration of this pathogenic progression is impossible without a global NTM surveillance network. Previously, *Mycobacterium avium* isolates recovered from patients’ lung samples were shown to match *M. avium* isolates recovered from the same patients’ household plumbing (4). Knowledge of the location, extent, and diversity of environmental NTM is important for both the vulnerable populations and the responsible clinicians.

Within the United States, the states of Florida and Hawai’i consistently show the highest per capita burden of NTM lung disease (LD) (5). In Hawai’i, LD cases are most commonly attributed to the *Mycobacterium avium* complex (MAC) in those identifying as Chinese, Korean, or Japanese, with a cumulative incidence rate of 247 cases/100,000 (2005–2019) (6). We previously reported widespread recovery of *Mycobacterium intracellulare* subsp. *chimaera* (henceforth referred to as *M. chimaera*) and *Mycobacterium abscessus* from a variety of environmental samples from Hawai’i, albeit in previously small sample-sized studies (7, 8). Prior studies from Hawai’i indicate multiple features linked to the prevalence of NTM in the environment, such as volcanic eruptions (9), soil with high moisture, iron, and hematite (10, 11), iron-rich environments that promote mycobacteria siderophore activity (12), vanadium metal in groundwater (13), and presence of NTM in stream water that percolates into aquifers, which is subsequently delivered to homes (14).

We hypothesize the breadth of NTM species found in the Hawaiian Islands is diverse and shows preferred niches and phylogenetic relatedness to other circulating NTM strains reported nationally and globally. Herein, we probed both built spaces and more natural, rural locations for NTM via one of the largest environmental sampling campaigns performed in Hawai’i, spanning the years of 2015 to 2019 with partial longitudinal sampling. We also examined 590 clinical samples from Hawai’i and other Pacific Islands that together provided an unprecedented opportunity to further understand the prevalence, ecological niches, and genomics of *M. abscessus* and *M. chimaera*.

Some of the results of these studies have been previously reported in the form of a poster (15).

## RESULTS

### Over 2,000 environmental samples were collected across the Hawaiian Islands

From 2015 to 2019, 2,334 unique environmental samples were collected across the islands of Kaua’i, O’ahu, Moloka’i, Maui, and Hawai’i Island. Samples were collected from both built sources (i.e., households and non-household sites, such as public buildings, schools) and natural sources (i.e., places with minimal human influence including streams, lakes, and forests) (Table S1). Four main sample types were collected across the islands: (i) water biofilms (e.g., “gunk” from showerhead surfaces, kitchen sink taps, bath/restroom sinks taps, ice machines, etc.), (ii) soil (collected from home gardens or natural areas), (iii) dust (swabs collected from indoor dusty areas of homes or outdoor locations), and (iv) natural water filters (i.e., water from streams passed through a filter).

### NTM were recovered from a quarter of environmental samples tested.

Of the 2,334 samples collected across Hawai’i, 541 (23%) were NTM culture positive. Samples were identified to the species level using partial *rpoB* gene Sanger sequencing (Table S2), and species that most commonly cause NTM LD (e.g., *M. abscessus, M. avium, M. chimaera, M. intracellulare*) were deemed “lung relevant” (LR). NTM positivity rates were stable across islands, suggesting the presence of NTM was independent of island age; e.g., Kaua’i (oldest in age) and Hawai’i Island (youngest) (16) (Table 1). An exception was Moloka’i, which yielded no NTM positive samples of the three received, likely due to the small sample size tested; thus, Moloka’i was removed from further analyses. When stratified by incubation temperature, 471 of the 541 (87%) samples cultured NTM at 30°C compared with 178 NTM positive samples cultured at 37°C (33%) (Fig. S1). Of the 471 samples that were NTM culture positive at 30°C, 115 were positive for LR NTM (24%) compared with 51 of the 178 NTM-positive samples that were culture positive at 37°C (29%).

**TABLE 1:** Description of Hawai’i environmental samples, any NTM species positivity

	Entire state	Kaua’i	O’ahu	Moloka’i	Maui	Hawai’i Island
Estimated age of island (in million years)	N/A^*a*^	4.7	2.6–3.0	2.0–1.8	1.2–1.5	0.6–0
Total no. of samples collected	2,334	763	765	3	386	417
Household: non-household: natural samples	804:1,142:388	225:387:151	305:303:157	0:0:3	149:196:41	125:256:36
Household: non-household: natural unique sites	150:525:123	48:161:57	55:137:24	0:0:3	25:96:18	22:131:21
NTM positivity	23% (541/2,334)	24% (185/763)	23% (177/765)	0% (0/3)	23% (87/386)	22% (92/417)
NTM positivity: Built environment	24% (472/1,946)	27% (164/612)	24% (144/608)	N/A	27% (93/345)	24% (90/381)
NTM positivity: Natural environment	18% (69/388)	21% (31/151)	27% (43/157)	N/A	7% (3/41)	10% (7/36)
Fisher’s exact test (*P*-value)	0.1384	0.1193	0.7617	N/A	0.0040	0.6825
Water biofilm NTM positivity	325/1,497: 22%	110/482: 23%	91/459: 20%	0/3: 0%	46/262: 18%	78/291: 27%
Soil NTM positivity	170/547: 31%	67/207: 32%	61/179: 34%	0/0: 0%	32/76: 42%	10/85: 12%
Dust NTM positivity	28/250: 11%	6/68: 9%	9/93: 10%	0/0: 0%	9/48: 19%	4/41: 10%
Stream water filter NTM positivity	18/40: 45%	2/6: 33%	16/34: 47%	0/0: 0%	0/0: 0%	0/0: 0%
Fisher’s exact test (*P*-value)	<0.0001	<0.0001	<0.0001	<0.0001	<0.0001	0.026

^*a*^ N/A, not applicable.

### NTM environmental hotspots identified on each island

**Fig 1:**
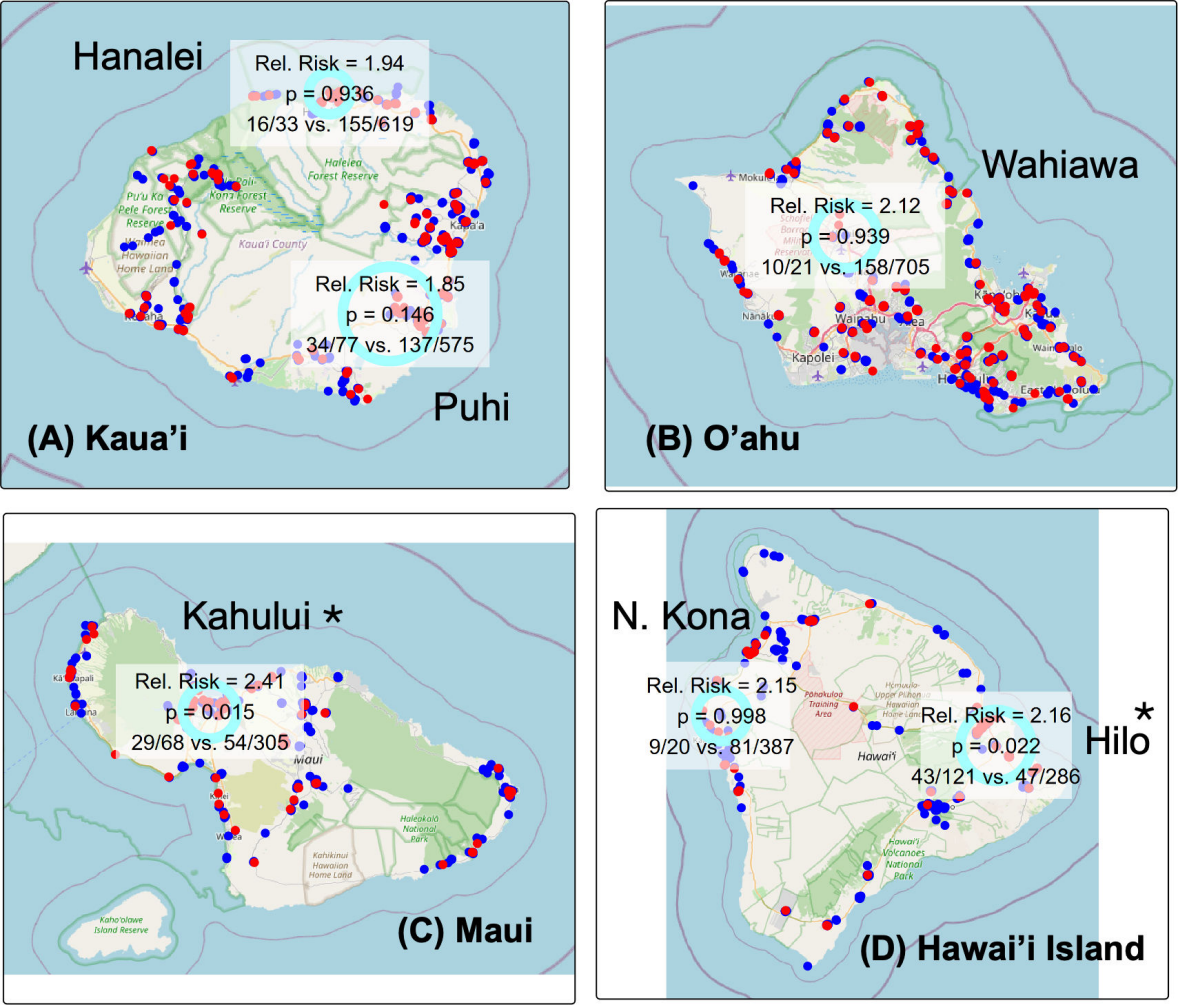
Hawai’i hotspots per island for environmental NTM recovery. Red and blue points indicate NTM culture-positive and NTM culture-negative locations, respectively, from (**A**) Kaua’i, (**B**) O’ahu, (**C**) Maui, and (**D**) Hawai’i Islands. Larger cyan circles highlight areas of higher relative risk for environmental NTM. Relative risk spots of *P* < 0.05 indicate a statistically significant hotspot, e.g., Maui and Hawai’i Islands. N/M vs P/Q indicate the fraction of samples within the hotspot that are positive (N/M) compared with the fraction of samples outside the hotspot that are positive (P/Q). The maps were created in R (v. 4.5.1) using the tmap package (v 4.1) and the tmaptools package (v. 3.3) with a base map from Open Street Map.

Hotspots were defined to be circular geographic regions inside which the ratio of positive samples was higher than the rate of positive samples outside the region. Hotspots were identified using a spatial scan statistics approach on the NTM culture results using the SaTScan program. Six environmental NTM hotspots were identified: (i) Hanalei in northern Kaua’i (Rel. Risk = 1.94, *P* = 0.936); (ii) Puhi in southeastern Kaua’i (Rel. Risk = 1.85, *P* = 0.146); (iii) Wahiawa on O’ahu (Rel. Risk = 2.12, *P* = 0.939); (iv) Kahului on Maui (Rel. Risk = 2.41, *P* = 0.015); (v); north of Kona on Hawai’i Island (Rel. Risk = 2.15; *P* = 0.998); and (vi) most of Hilo and Hawaiian Paradise Park on Hawai’i Island (Rel. Risk = 2.16, *P* = 0.022). These hotspots include some of the larger population centers of Hawai’i. However, only the Kahului and Hilo hotspots were statistically significant (Fig. 1A through D).

For *M. abscessus* (Fig. S2), only one hotspot was detected, centered on the Pearl Harbor on O’ahu (Rel. Risk = 3.43, *P* = 0.314). For *M. chimaera* (Fig. S3), two hotspots were detected, one in Puhi, Kaua’i (Rel. Risk = 8.35, *P* = 0.004), and the other in Pearl City, O’ahu (Rel. Risk = 4.48, *P* = 0.789). Of these species-specific hotspots, only the Puhi hotspot was statistically significant.

### Built and natural environments show similar likelihood of NTM recovery

We next analyzed the recovery rate for NTM from 2,334 samples obtained from human-built environments compared with samples collected from natural, more pristine locations. Generally, the frequency of NTM recovery between built and natural environments was not statistically different per island (Table 1). The exception was Maui where built environments were significantly more likely to harbor NTM compared with natural samples tested.

### Soil is more likely to harbor any NTM species compared with water biofilms and dust

NTM species in the context of the four sample types obtained were compared: (i) water biofilms, (ii) soil, (iii) dust, and (iv) 0.2-μm water filters collected after filtering freshwater from streams. When studied by sample type, 31% (170/547) of soil harbored any species of NTM, followed by water biofilms (22%, 325/1497), and dust (11%, 28/250) (Table 1). Notably, while stream water filters show a high NTM positivity rate (45%, 18/40), there were also appreciably lower total samples than the other sample types, and stream samples were only sparely collected from two of the four islands. Taking this into account, NTM were most prevalent in soil though the degree of this prevalence varied by island. For example, soil from the Big Island was significantly less likely to be NTM culture positive compared with Maui soil (*P* < 0.0001). NTM-negative samples varied across the islands and sample types (Table 1), with soil from Hawai’i Island built environments (e.g., home gardens) (10%, 8/82), Maui showerheads (16%, 14/88), and O’ahu sinks (19%, 34/176) demonstrating lower NTM positivity rates compared with their other island counterparts.

### Absence of a dominant environmental NTM species

**Fig 2:**
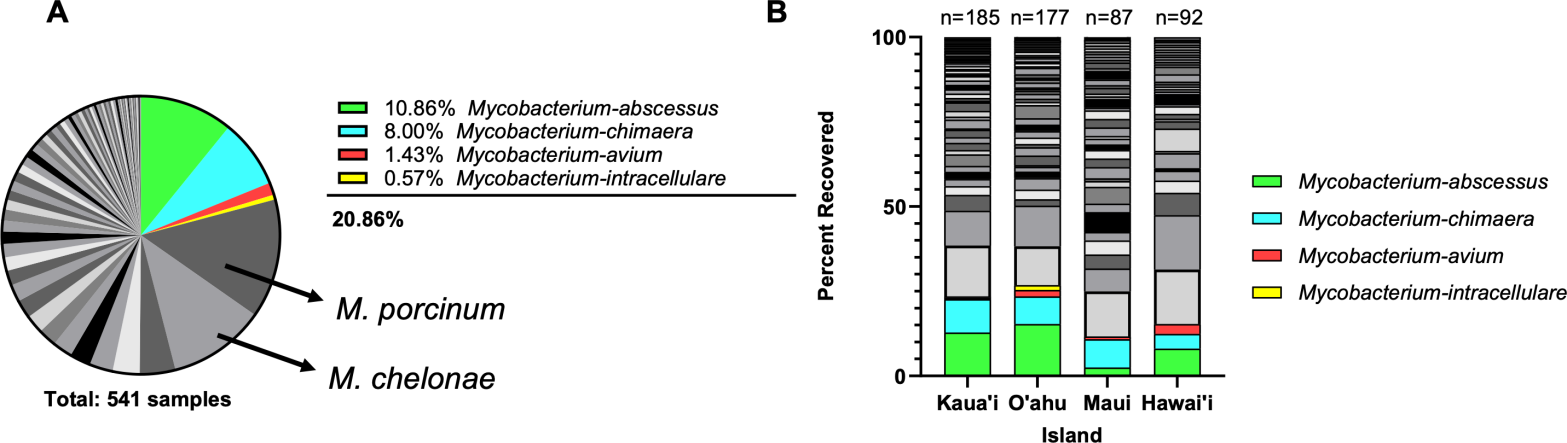
Representation of lung relevant (LR) NTM species among 541 NTM culture-positive environmental samples and stratified by island. (**A**) Proportion of the species from the 541 NTM-positive environmental samples collected across the State of Hawai’i that were LR species (colored pies). Non-LR species are shown in gray scale. (**B**) Composition of LR NTM species among the NTM positive environmental samples represented by island distribution. Non-LR NTM species are shown in gray scale.

In total, 74 different NTM species were identified from the 541 positive samples. A portion (2%, 58/2,334) was not identifiable using *rpoB* sequencing and was deemed putative novel NTM and excluded from further analysis (Table S2). Most NTM-positive samples cultured only one NTM species (77% 417/541). The proportion of multiple species identified from a single sample was 2 (18% 96/541) and 3 species (4% 23/541). Less than 1% cultured 4–5 species (3/541 and 2/541, respectively) (Table S3). No single species was overrepresented across the positive samples tested. Statewide, the most frequently recovered species identified from any sample were *Mycobacterium porcinum* 13.9% (97/700)*, Mycobacterium chelonae* 11.3% (79/700)*, M. abscessus* 10.9% (76/700), and *M. chimaera* 8% (56/700) (Table S1; Fig. 2A).

We next categorized each NTM as either a lung relevant (LR) species (i.e., one of the four species most commonly reported to cause human LD) or a “non-LR” species (i.e., low likelihood of causing significant human LD beyond colonization). Using this stratification, 21% of the isolates recovered from each sample were LR (Fig. 2A). LR NTM were represented in a minority of samples tested from Kauai (24%), O’ahu (27%), Maui (12%), and Hawai’i Islands (15%) (Fig. 2B). Despite ~100 mile separation between islands, *M. avium*, *M. abscessus,* and *M. chimaera* were recovered from all islands, albeit in different proportions. While *M. intracellulare* and *M. avium* represent the prominent LD-causing species on the U.S. continental mainland, they were rare in Hawai’i environments, with *M. avium* and *M. intracellulare* present in 0.4% (10/2,334) and 0.2% (4/2,334) of samples, respectively. Aside from a single dust sample, *M. avium* was primarily recovered from water biofilm samples (*n* = 9 samples), while *M. intracellulare* was only recovered from three biofilm samples and one water filter (Table S2). While their characterization was beyond the scope of this study, 58 environmental and 13 lung isolates were identified as unspeciated/novel.

### Broadly, water biofilms are a preferred environment for LR NTM, and household showerheads were consistently positive for LR NTM across longitudinal samples

**Fig 3:**
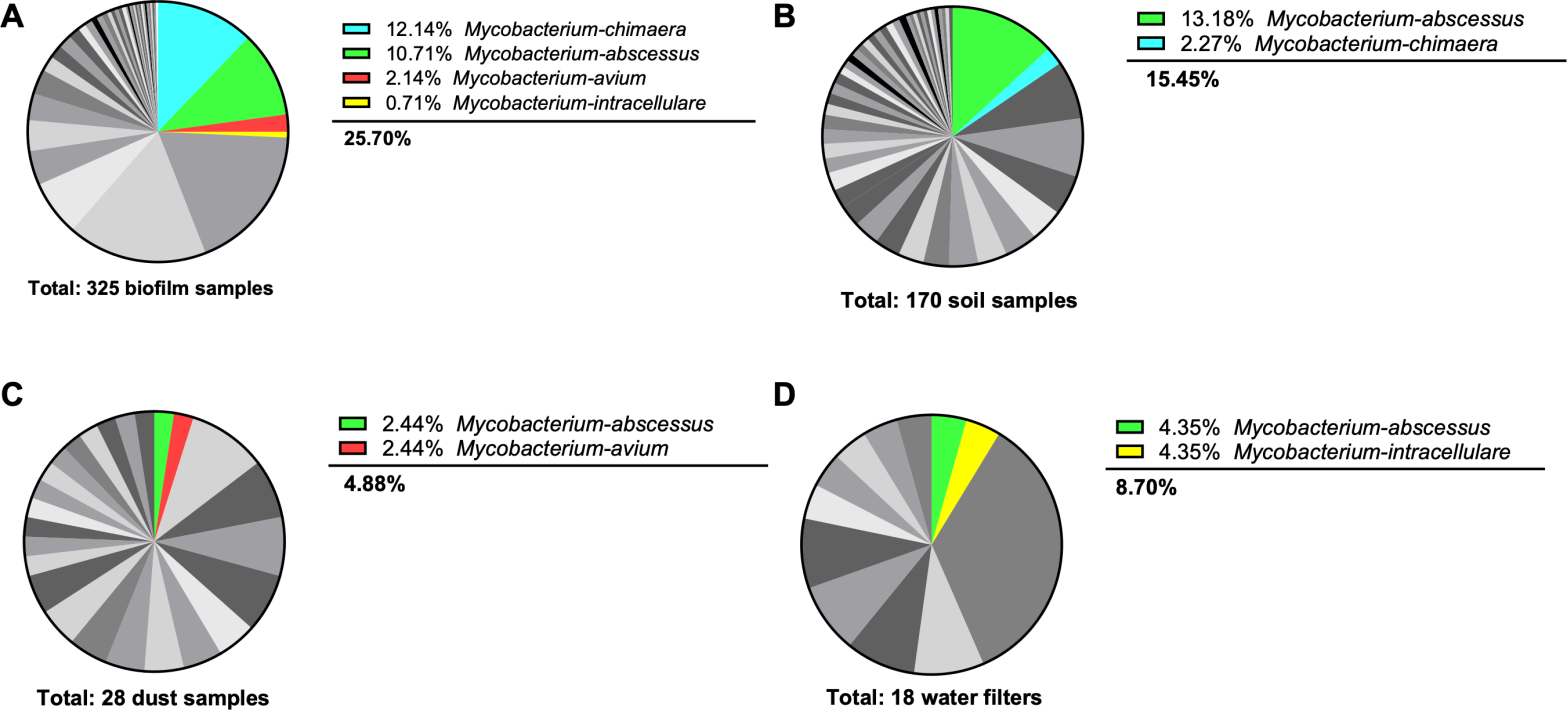
LR NTM species show preferred environmental niches and prefer to colonize water biofilms***.*** Species diversity of the NTM-positive environmental water biofilm (**A**), soil (**B**), dust (**C**), and water filter (**D**) samples collected across Hawai’i. Non-LR species are shown in gray scale.

The samples containing LR NTM occurred in the following order of frequency: water biofilms (26%) > soil (15%) > water filters (9%) > dust (5%) (Fig. 3). LR species were more likely to be found in water biofilms than non-LR species, with an odds ratio of 2.30 (95% CI: 1.52, 3.55; *P* = 0.0000315). *M. abscessus* was the only LR NTM recovered from all four environmental categories tested.

**Fig 4:**
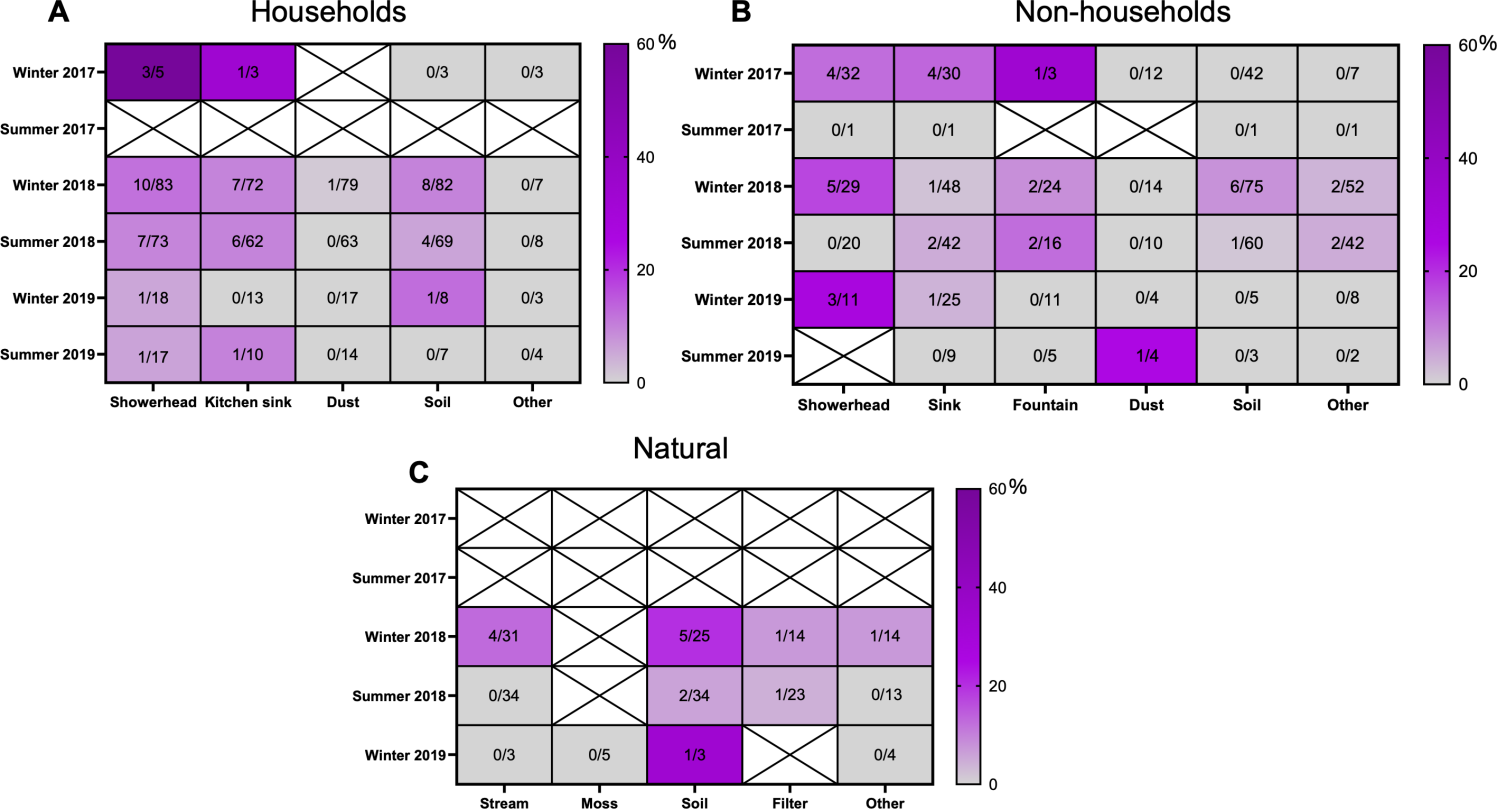
LR NTM species are not consistently recovered over time or from similar sources*.* The percentage of sample types from unique households (**A**), non-household locations (**B**), and natural sources (**C**) that recovered LR NTM species are shown as a gradient from 0% (gray) to 60% (dark purple). White crossed boxes indicate no samples were collected for that sampling time. Natural samples were not collected Summer 2019.

To assess whether NTM culture positivity changed over time per sample type across the longitudinal samples collected, we tabulated the number of unique environmental niches among household, non-household, and natural samples across the Winter 2017–Summer 2019 sampling periods that were NTM culture positive for LR NTM (Fig. 4) and non-LR NTM (Fig. S4). While longitudinal sampling was not performed for each timepoint for all samples collected, household showerheads were the only environmental niche that was consistently positive for LR NTM over five time periods during which household sampling was performed (Fig. 4A through C). In parallel, LR NTM were identified from natural soils in each of the three time periods from which natural samples were collected (Winter 2018–Winter 2019, Fig. 4C). In contrast, non-LR NTM were often common colonizers of many diverse household, non-household, and natural area niches (Fig. S4A through C).

### Compared with SGM species, RGM are broad generalists capable of colonizing diverse environmental niches

**Fig 5:**
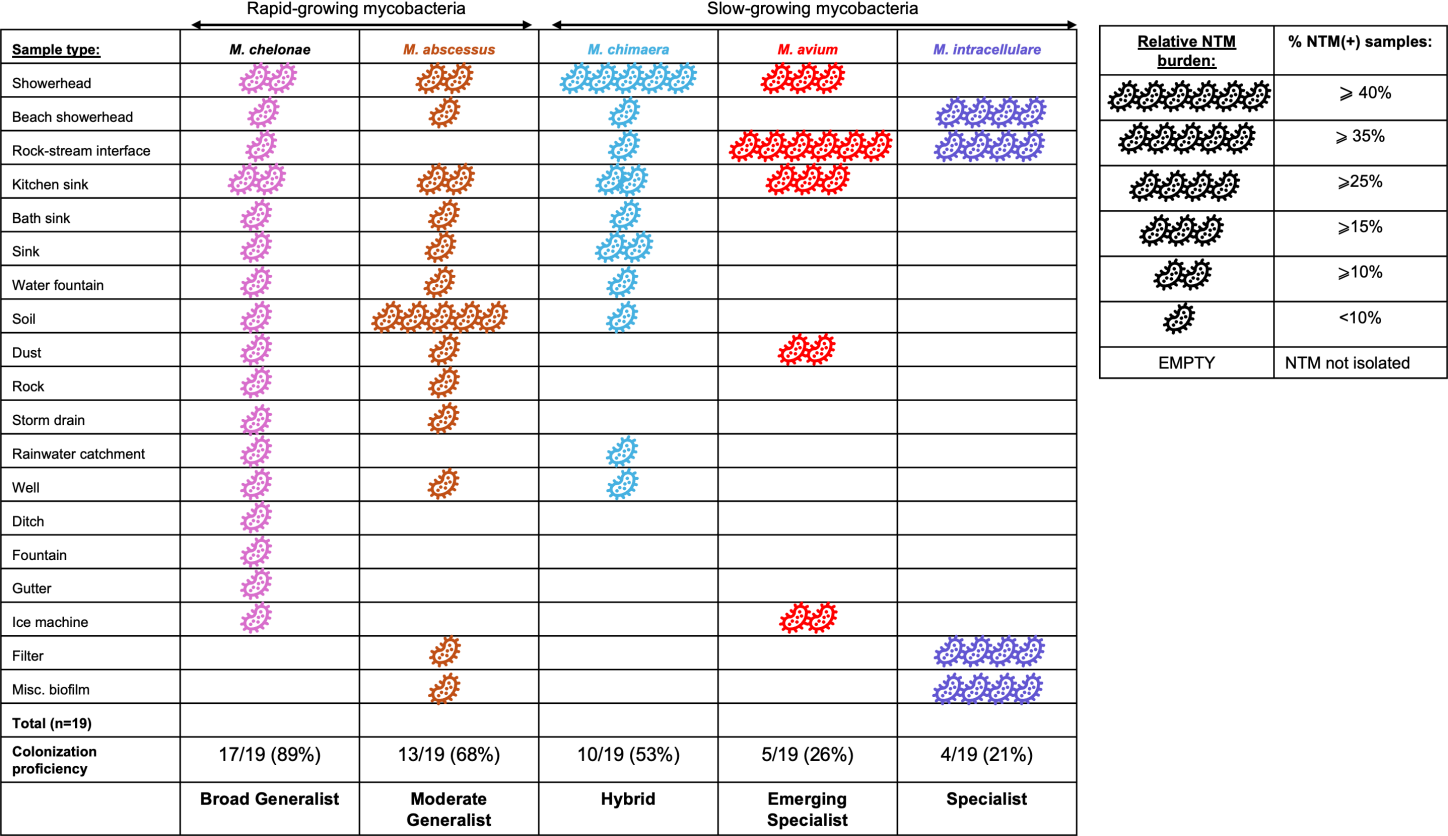
Capacity to colonize diverse environments is species specific*.* In total, 19 different types of environmental samples were collected throughout the study from which rapid- and slow-growing NTM were identified, and proportions of positive samples were ranked (key). Based on the proportion of samples in which a particular species was found, NTM were categorized as broad generalists (highly adaptable) to specialists (better adapted to specific niches)—ecological terms used to categorize specific NTM species capacity to tolerate and colonize environments.

To elucidate the environmental adaptability of individual species and the ecological diversity patterns of LD-causing NTM in Hawai’i, we tabulated the relative abundance for a set of species across the 19 different types of environmental samples tested (Fig. 5). RGM *M. chelonae* was recovered from 17/19 (89%) sample types tested, befitting a broad generalist. *M. abscessus* was categorized as a moderate generalist colonizing 68% (13/19) of environments tested. In comparison, SGM MAC were ecological specialists only recovered from 21% (4/19) environments tested, e.g., *M. intracellulare*.

### *M. chimaera* was the most frequently identified species in lung samples

To determine whether NTM found in the Hawai’i environment were representative of the NTM found in Hawai’i respiratory samples, 590 isolates recovered from sputum of de-identified Hawai’i patients with suspected mycobacterial infection were characterized by *rpoB* gene sequencing. *M. chimaera* was the most common species recovered at 40% (238/590), followed by *M. abscessus* (20%, 118/590) (Table 2).

**TABLE 2:** *M. chimaera* is the most frequent NTM species in lung samples^*a*^

Species	Number of isolates	Percentage of isolates
*M. chimaera*	238	40.34%
*M. abscessus*	118	20.00%
*M. avium*	59	10.00%
*M. fortuitum*	41	6.95%
*M. porcinum*	30	5.08%
*M. intracellulare*	18	3.05%
*M. yongonense*	14	2.37%
*M. timonense*	11	1.86%
*M. chelonae*	9	1.53%
*M. kubicae*	7	1.19%
*M. gordonae*	6	1.02%
*M. lentiflavum*	6	1.02%
*M. simiae*	6	1.02%
*M. conceptionense*	4	0.68%
*M. paragordonae*	3	0.51%
*M. senegalense*	3	0.51%
*M. marseillense*	2	0.34%
*M. paraintracellulare*	2	0.34%
*M. parascrofulaceum*	2	0.34%
*M. arosiense*	1	0.17%
*M. canariasense*	1	0.17%
*M. celatum*	1	0.17%
*M. elephantis*	1	0.17%
*M. gastri*	1	0.17%
*M. kansasii*	1	0.17%
*M. kumamotonense*	1	0.17%
*M. mucogenicum*	1	0.17%
*M. phocaicum*	1	0.17%
*M. scrofulaceum*	1	0.17%
*M. shigaense*	1	0.17%
TOTAL	590	100%

^*a*^ A total of 590 unique lung derived NTM isolates from de-identified Hawai’i patients were subjected to partial *rpoB* gene sequencing for NTM species identification.

### Hawai’i environmental *M. abscessus* commonly belongs to dominant circulating clone 1

**Fig 6:**
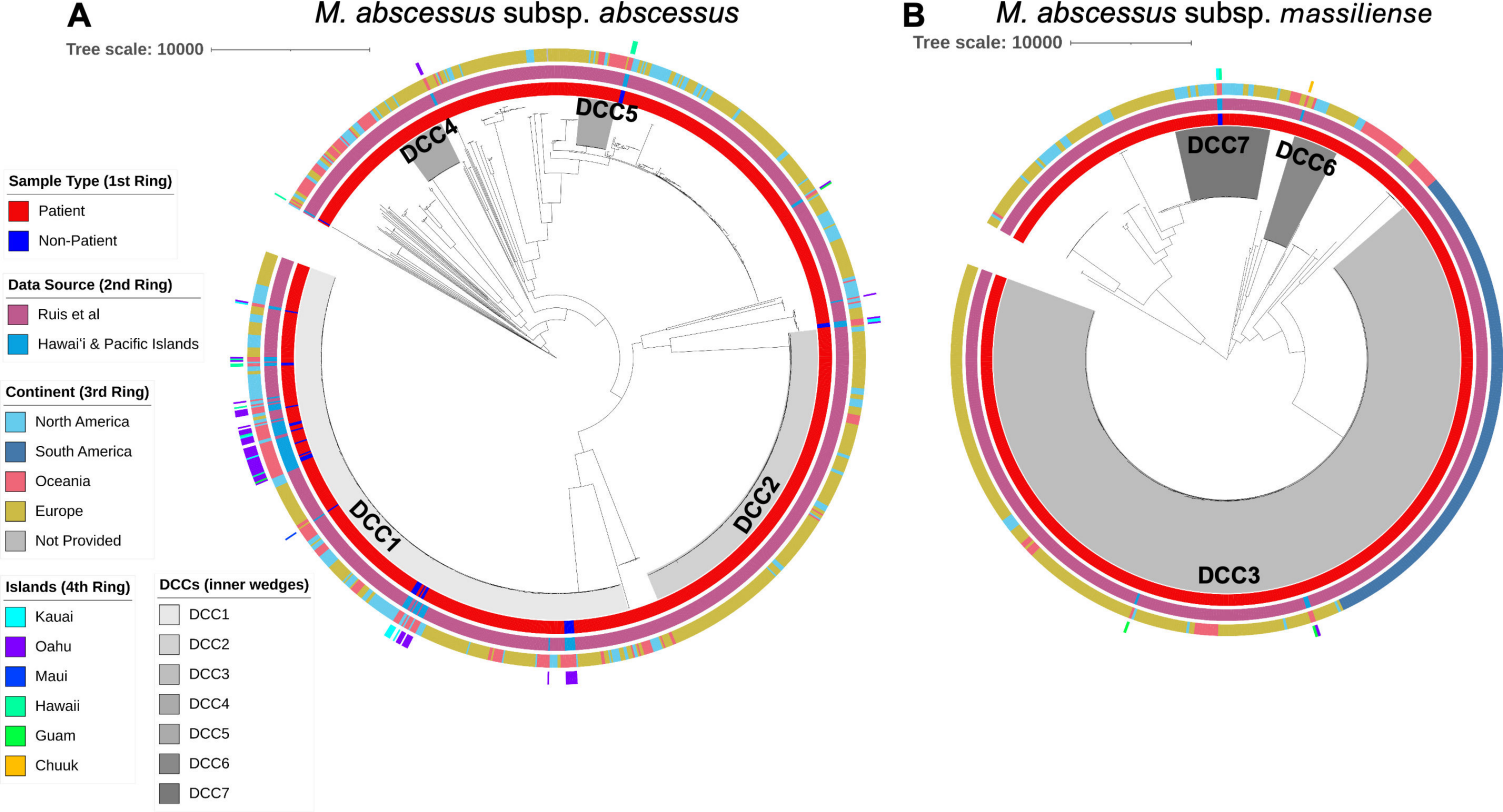
Hawai’i environmental *M. abscessus* commonly belongs to dominant circulating clone 1*.* Phylogenetic analysis of two subspecies of *M. abscessus*, combining the (18) data set with Hawai’i isolates from the present study. In both plots, the clades representing established DCC groups are labeled and shaded in gray; the first ring indicates the study of origin for each isolate, and the second ring labels the continent where each isolate was collected. (**A**) A phylogenetic tree showing the relationship between Hawai’i isolates (*n* = 80) and existing isolates for *M. abscessus* subsp. *abscessus* (*n* = 1,098). (**B**) A phylogenetic tree showing the relationship between Hawai’i isolates (*n* = 6) and publicly available isolates for *M. abscessus* subsp. *massiliense* (*n* = 621).

Next, we investigated the relationship of Hawai’i *M. abscessus* to the dominant circulating clones (DCCs) (17) by constructing phylogenetic trees for *M. abscessus* subsp. *abscessus* and *M. abscessus* subsp. *massiliense,* combining our data with publicly available genomes (18). Most *M. abscessus* subsp. *abscessus* clustered with DCC1 (84%; 67/80), while the rest formed clades with sub-DCC level clusters (*abscessus* 2, 12, 14, 16) (Fig. 6A). All our *M. abscessus* subsp. *massiliense* (100%; 6/6) clustered within established DCCs: three in DCC3, two in DCC7, and one in DCC6 (Fig. 6B).

The inducible macrolide-resistance *erm(41*) gene has been shown to exhibit evidence of positive selection in *M. abscessus* subsp. *abscessus* (19). Thus, we examined *M. abscessus* subsp. *abscessus* from our environmental collection for *erm(41*) genotypes at polymorphic positions. The majority (84%, *n* = 67/80) showed V80I mutations; isolates containing this mutation clustered within DCC1. Because the C allele at position 28 confers macrolide sensitivity (19), position 28 was further examined. Of 80 isolates from our collection, five (6%) carried the C allele and clustered outside of known DCCs.

### Pacific Island circulating clusters of *M. chimaera* are distinct from previously reported heater-cooler *M. chimaera*

**Fig 7:**
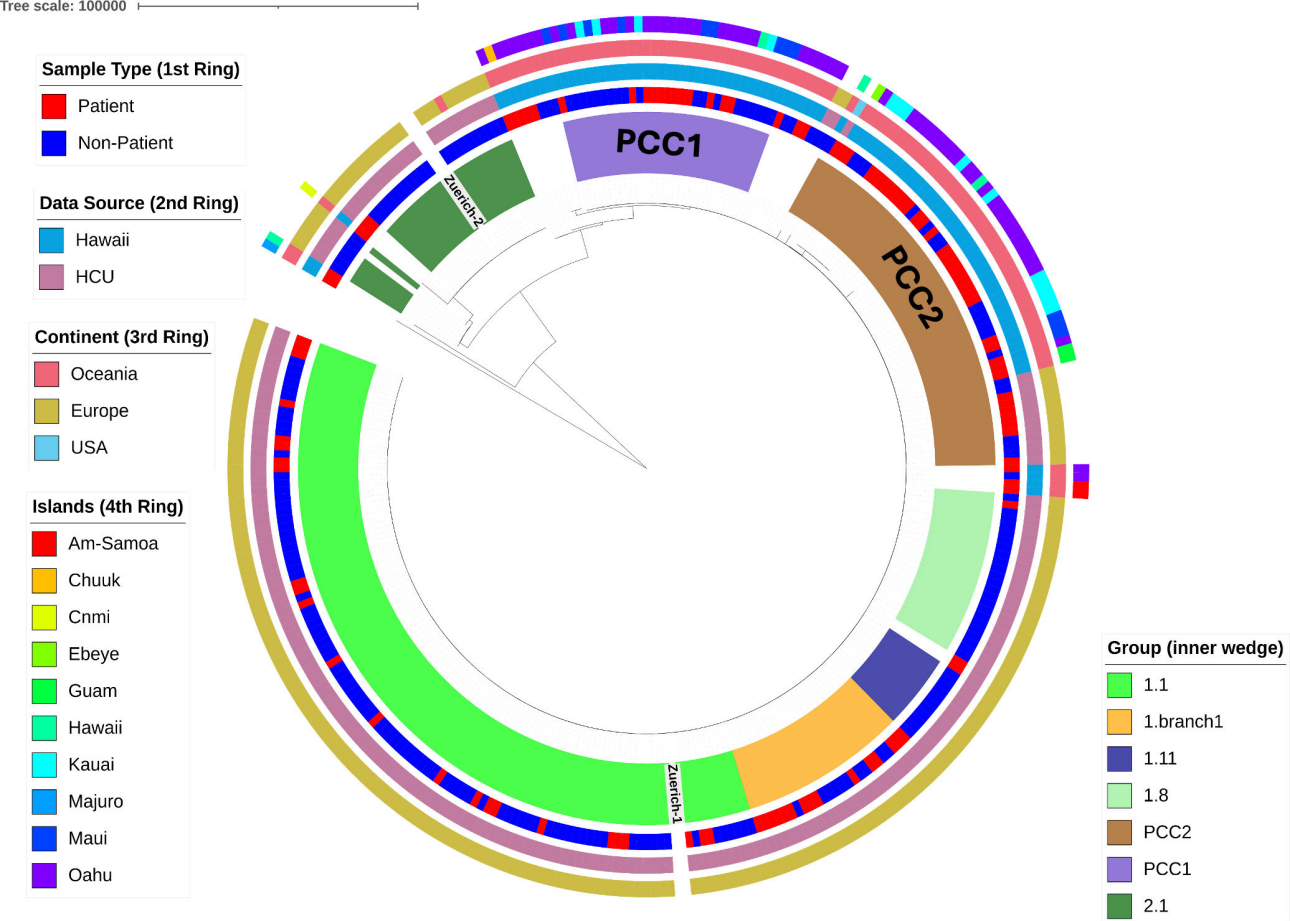
Pacific Island circulating clusters of *M. chimaera* are distinct from previously reported heater-cooler *M. chimaera*. Phylogenetic analysis using an integrated *M. chimaera* data set from an HCU outbreak study (*n* = 223) and Hawai’i/Pacific Island *M. chimaera* isolates (*n* = 92). Leaves are color-coded according to the phylogenetic groups from the previously defined HCU outbreak study and the newly identified Pacific Island Circulating Cluster 1 and 2 (PCC1 and PCC2). Annotations in the first ring indicate patient or non-patient sources of isolates. Annotations in the second ring indicate the names of the project. Annotations in the third and fourth rings indicate geographic locations of sample collections.

Phylogenetic analysis of *M. chimaera* using a combined data set of Hawai’i/Pacific Island isolates and data from previous publications of heater-cooler unit (HCU) outbreaks (20) reproduced the grouping pattern observed in the HCU outbreak genomic study. Of note, most Hawai‘i and other Pacific Island isolates grouped separately from HCU-associated samples. Clade analysis revealed two novel groups we designate as Pacific Island Circulating Clusters 1 and 2 (PCC1 and PCC2) (Fig. 7; Fig. S5). PCC1 was composed exclusively of isolates from Hawai‘i and the Pacific Islands, indicating a distinct regional lineage. The median pairwise distance is 18 SNPs in PCC1 (range 0–37). PCC2 included a majority of Hawai‘i and Pacific Island isolates, along with a subset of samples from Europe and the continental United States. The median pairwise SNP distance in PCC2 was 56 (range 0–103). Most PCC2 isolates (66.7%, 36/54) were derived from lung specimens, while a smaller portion of lung samples (38.5%, 15/39) were in PCC1 (Fig. 7; Fig. S5).

## DISCUSSION

In this study, our goal was to learn more about the diversity of environmental NTM within the landscape of Hawai’i for two overarching purposes. First, the high number of LD cases reported in Hawai’i underscores the pressing need to elucidate how environmental NTM diversity mirrors or does not mirror those species responsible for LD in a U.S. geographic hotspot. Second, we aimed to extend the current knowledge of where patients may be exposed by conducting one of the largest environmental sampling campaigns for NTM performed to date, applying a community science network to perform diverse niche sampling across the most populated Hawai’i islands in a longitudinal way. Culture media produced viable isolates for species identification using *rpoB* gene sequencing, and isolates identified as LR were further confirmed using WGS.

Large-scale environmental sampling campaigns for NTM are limited. Gebert et al. performed a culture-independent 16S rRNA gene sequencing survey of 606 U.S. household showerheads, finding a high prevalence of *Mycobacterium* corresponding to U.S. geographic regions with the highest reported NTM LD incidence (21). Walsh et al. conducted soil sampling (143 U.S. locations using 16S rRNA and *hsp65* sequencing) and demonstrated that MAC isolates were more frequently associated with wet and acidic soil (22). To the best of our knowledge, our collection of 2,334 diverse samples from not just households but also non-households and natural sites, including 1,498 water biofilms, 547 soils, 250 dust, and 40 water filters, is one of the largest of its kind performed to date.

In our prior report of household plumbing samples in Hawai’i and soil collected between 2012 and 2013, the positivity rate was 44% (75/172) (7), with *M. chimaera* the most frequently identified NTM. *M. chimaera* was also the most frequently identified NTM (45%, *n* = 98) among 218 lung samples from Hawai’i patients (8). We verified *M. chimaera* predominance in an additional set of 590 Hawai’i lung samples (Table 2). Nearly a quarter (23%, 545/2,334) of the environmental samples reported in the current work were shown to be NTM positive. Lower recovery of NTM in the current study compared with our previous work may be the result of studying a smaller sample set of 172 (8) versus 2,334 samples in the current study and an increased sample range represented in our current work. Based on outcomes shown in Fig. 5, we deduce *M. abscessus* and MAC show varying colonizing niches. The consistency and reliability in recovering LR *M. abscessus* and *M. chimaera* suggest that these opportunistic pathogens are endemic in these tropical islands.

Our phylogenomic analyses revealed that 100% of Hawai’i *M. abscessus* subsp. *massiliense* environmental isolates (*n* = 6/6) clustered within DCC3 (*n* = 3), DCC7 (*n* = 2), and DCC6 (*n* = 1) (Fig. 6B). We discovered the majority of Hawai’i *M. abscessus* subsp. *abscessus* isolates clustered within DCC1, with the remaining isolates scattered within 2, 12, 14, and 16 sub-DCC level subclades (Fig. 6A). It has been postulated that the emergence of DCCs is a recent event due to the high level of genetic relatedness among *M. abscessus* isolates globally (18). Alignment of Hawai’i *M. abscessus* with DCC1 parallels prior reports from most lung isolates from people with cystic fibrosis (CF) belonging to DCC1 (17).

Yang et al. recently reported that the *erm(41*) V80I mutation is a *M. abscessus* subsp. *abscessus* mutation under positive selection, affecting 50.5% of strains and not associated with increased antibiotic resistance (19). In our collection, the percentage of isolates with the V80I mutation was higher at 84%. In the same study (19), macrolide resistance associated with *erm(41*) 28T and macrolide sensitivity from a 28C transition was investigated. Among our 80 Hawai’i *M. abscessus* subsp. *abscessus* isolates, only five (6%) carried the C allele and clustered outside of known DCCs, suggesting that most of our isolates may experience selective pressure to maintain macrolide resistance. The significance of these mutations remains to be elucidated.

Freshwater drinking systems remain under-characterized with a paucity of environmental NTM reference genomes available (23). However, WGS approaches have been applied to interrogate drinking water as a potential *M. abscessus* reservoir (24). Thomson et al. analyzed of 58 water isolates and 231 respiratory isolates and reported water is likely involved in NTM transmission from the environment to susceptible hosts. From the healthcare-associated links in transmission (HALT) investigations of *M. abscessus* outbreaks among CF patients, person-to-person transmission of *M. abscessus* from healthcare environments to CF patients is an uncommon to rare occurrence (25, 26).

In the context of *M. chimaera*, the HALT project suggests the possibility of healthcare associated environmental acquisition for people with CF (27). Other visible studies of environmental acquisition of *M. chimaera* are highlighted by the heater-cooler unit (HCU) outbreaks originally reported in Zurich (28), Europe, Australia, and the United States (29). In France, 72% (26/36) of outbreak *M. chimaera* HCU isolates belonged to the epidemic cluster (30). To contextualize the *M. chimaera* isolates from our study, comparisons to HCU isolates were made from which three novel results were found. First, *M. chimaera* from Hawai’i and other Pacific Islands were not directly related to the ZUERICH-1 *M. chimaera* HCU cluster. The minimal SNP distance from PCC1 to Zuerich-1 was 32, and PCC2 to Zuerich-1 was 25. Second, *M. chimaera* from Hawai’i and other Pacific Islands were distinct from the European ZUERICH-2 environmental cluster. Third, a major clade (Clade 2) consisting mostly of clinical samples from Hawai’i and European sites suggests common mechanisms of pathogenicity or transmission across geographically distinct regions. To separate these from *M. chimaera* HCU, we designate the environmental *M. chimaera* from Hawai’i and other Pacific Islands clustering into their own distinct group as Pacific Island Circulating Cluster 1 (PCC1) and Hawai‘i and Pacific Island respiratory isolates clustering separately as PCC2 (Fig. 7).

In addition to the novel discovery of PCC1 and PCC2, a unique feature of this study was the extended timeframe for environmental sample collection (2015–2019), which allowed us to perform limited repeat sampling. We recognize an important caveat to our longitudinal community-based sampling strategy was that high numbers of samples could not be equivalently collected in all six sampling periods across the years, and sampling was not performed at every timepoint. Nonetheless, non-LR NTM species were found to consistently colonize diverse niches across households, non-households, and natural sites that spanned across the sampling years (Fig. S4). By comparison, our results provide additional evidence supporting LR NTM (i.e., *M. abscessus* and MAC) have preferred niches. That is, LR NTM were routinely identified from household showerheads within each of the five time periods during which home sampling was performed. LR NTM were also commonly identified from home kitchen sinks across four of the five sampling periods (Fig. 4A). By comparison, LR NTM were not recovered from any of the home dust samples tested. Outside of the home, non-household sampling of similar water biofilm sources, dust, and soil revealed inconsistent recovery of LR NTM through time (Fig. 4B). Taken together, these data support prior reports of household water and water distribution systems as likely sources of NTM exposure (21, 24, 31–33). A novel aspect of this study was including non-household sampling during the same periods as home sampling. LR NTM were recovered from non-household samples sporadically (Fig. 4B). It is likely susceptible individuals interact with non-household niches such as beach showerheads, public bath sinks, and water fountains only momentarily making transient exposures unlikely. Rather, the preponderance of LR NTM from household water sources and the extended time patients spend in their homes may increase the likelihood of possible exposures. This inference is supported by a separate study where we performed longitudinal tracing of environmental NTM from a single home in Hawai’i across a 12-year timeframe (34). Despite living in the home for more than four decades, the homeowner developed *M. chimaera* LD only after a bronchiectasis diagnosis. Using WGS and comparative phylogenetic analyses, we genomically matched the homeowner’s lung-derived *M. chimaera* isolates to the *M. chimaera* recovered from the home across the 12 years, including the homeowner’s showerhead and kitchen sink.

There are limitations to the current study. First, we relied upon opportunistic community-based sample collection, which may not fully represent the environmental niches of Hawai’i. While this approach allowed access to a wide range of geographic locations that would have been difficult for our team to reach over 5 years, it could have introduced variability in sampling techniques, handling, and storage conditions. Despite our effort to perform widespread sample collection, many more areas were, of course, not sampled than were sampled, simply because comprehensive sampling of every environment is impossible. Second, we did not account for the effects of seasonal variation on recovery rates and the diversity of NTM due to the changes in environmental conditions over time. Third, NTM in nature may be influenced by specific factors in their occupying niche, factors not possible to reproduce in the laboratory. Our reliance on culture-based methods may not fully represent the NTM in each collected sample. Fourth, our study included clinical samples but lacked integration of patient data, limiting our ability to correlate environmental NTM with clinical outcomes or draw definitive conclusions about specific public health impacts of environmental NTM. Finally, genomic analyses were limited to *M. abscessus* and *M. chimaera*, not applied to all NTM. In particular, species identification for isolates not belonging to *M. abscessus* or *M. chimaera* were based on partial *rpoB* sequencing and were not confirmed by whole genome sequencing, which may cause for the failure to discriminate between closely related NTM species.

In summary, our exploratory and comparative phylogenomic studies of NTM from the geographic hotspot of Hawai’i provides additional insight into the prevalence, ecological niches, and genomics of *M. abscessus* and *M. chimaera*. Overall, nearly one in four environments sampled tested positive for NTM. But in terms of *M. abscessus* and MAC, our data further strengthens the argument that homes water systems as modifiable targets for prevention. Our discovery of distinct locally circulating strains (e.g., PCC1, PCC2) shows that NTM epidemiology may be region specific, requiring tailored surveillance and public health strategies. Future studies to better understand environmental expansion patterns and pathoadaptation strategies of endemic NTM in other similar geographic hotspots are needed as potential ranges for NTM have already been forecasted to surge in Hawai’i as temperatures increase (35). We predict discovery of more novel species, clades, and clusters of NTM are likely to be discovered with increasing clinical consequences for susceptible individuals.

## MATERIALS AND METHODS

### Large-scale NTM environmental sampling, isolation, and identification

From 2015 to 2019, high school and university level students from 11 schools across Hawai’i as well as community volunteers used opportunistic sampling to collect 2,334 unique environmental samples (1,946 and 388 samples from built and natural environments). After collection, samples were stored at 4°C or −20°C for 1–30 days before shipment. Of the built environments, 1,142 samples were collected from 525 unique non-households and 804 samples from 150 unique households (Table S1). Environmental samples were processed within 7–270 days post receipt. Samples were cultured for NTM using standard microbiological approaches and characterized by partial *rpoB* gene Sanger sequencing (supplemental methods) (Table S2) (8). Isolates highly matched (greater than 90% BLAST identity and coverage) to uncharacterized mycobacteria were deemed as potential novel NTM but were excluded from analyses because their characterization was beyond the scope of this study.

### Map generation and hotspot detection

Locations of NTM+/−, *M. abscessus*+/−, and *M. chimaera*+/− samples were mapped to each of the four sampled islands (Kauai, O’ahu, Maui, and Hawai’i Island) using the OpenStreetMap and tmap packages (36) in the R language (37). Sample was colored red for recovery and blue for non-recovery. The SaTScanTM program was used to detect geographic hotspots assuming a dichotomous outcome (38). SaTScanTM reported the relative risks and *P*-value for each hotspot. Only hotspots > 1 km in diameter and including at least four positive samples were mapped since smaller hotspots included only a handful of sampling sites.

### Definition of lung relevant (LR) NTM species

For the described analysis, NTM were classified as lung relevant (LR) if belonging to MAC, i.e., *M. avium* subsp. *hominissuis* (*M. avium*), *M. intracellulare* subsp. *intracellulare* (*M. intracellulare*), and *M. intracellulare* subsp. *chimaera* (*M. chimaera*) (39, 40) and included *M. abscessus* (41) as the top four most common LD causing species (42).

### Respiratory NTM samples

Randomly selected, de-identified NTM respiratory samples (*n* = 590, September 2014 to January 2016) recovered from the sputum of Hawai’i patients suspected of mycobacterial LD were obtained. Because the samples were delinked from the originating patient sources, private health information was not available, including if each patient met current American Thoracic Society/Infectious Disease Society of America (ATS/IDSA) diagnostic criteria for NTMLD. This activity was determined as non-human subject research by the Kaiser Permanente Hawai’i Institutional Review Board. Isolates were subjected to *rpoB* gene sequencing and categorized as LR after whole genome sequencing (WGS).

### Comparative phylogenomics

Genomic DNA was extracted from pure isolates as published (43). Sequencing libraries were constructed using Illumina reagents Nextera XT, DNA Flex, or DNA Prep and sequenced on the MiSeq, HiSeq, NextSeq, or Novaseq instrument using paired end 2 × 300 bp or 2 × 250 bp at a depth of 40× coverage across each genome.

Adaptor sequences were trimmed using SeqPurge (44). To verify species identity and check for contamination, kraken2 version 2.1.3 was used (downloaded May 2024) (45). Samples with >90% of classified reads belonging to *M. abscessus* reported by kraken2 were retained. *M. chimaera* samples were retained if they met the following criteria reported by kraken2 analysis: (i) >50% identity as MAC, (ii) highest ranking as matching to *M. intracellulare* with all other species matching <2%, and (iii) highest ranking as matching to *M. chimaera* at subspecies level with all other subspecies matches <2%. The species of samples identified by kraken2 were verified via bowtie2 (version 2.5.4) (42) alignment using relevant reference genomes (*M. abscessus* ATCC 19977 for *M. abscessus* and *M. chimaera* DSM44623 for *M. chimaera*). We selected samples that showed >75% coverage of the reference genomes, generating a final set of 87 *M. abscessus* and 92 *M. chimaera* for downstream analyses.

SortSAM from gatk package (version 4.6.1) (46) was applied for sorting and file manipulation and bcftools (version 1.21) (47) for variant calling and consensus fasta generation. To separate *M. abscessus* samples by subspecies, fastANI was used (version 1.34) (48) using the references (GCF_000069185.1, GCF_001792625.1, and GCF_003609715.1) for *M. abscessus* subsp. *abscessus*, *massiliense*, and *bolletii*, respectively.

To place isolates in the context of global studies, we downloaded data from Ruis et al. (18) for *M. abscessus* and from van Ingen et al. (29) for *M. chimaera*, and parsnp (version 2.1.3) (49) was used to generate core genome alignments and gubbins (version 3.4) applied to remove predicted recombination, using FastTree (2.1.11) to generate the initial phylogenetic trees and raxmlHPC (8.2.12) to construct the final phylogenetic trees. Final trees consisted of Hawai’i *M. abscessus* subsp. *abscessus* (*n* = 80), *M. abscessus* subsp. *massiliense* (*n* = 6), and *M. chimaera* (*n* = 93). Phylogenetic trees were generated using iTOL (version 7.2) (50). The clades in the *M. chimaera* phylogenetic tree were defined by converting the tree to a cladogram (Fig. S5) and applying a branch-height cut to partition it into monophyletic groups. The cut height was chosen empirically as the level that best separated previously published groups in the HCU *M. chimaera* study (29).

### Statistical analyses

Fisher’s exact test was used to calculate probabilities of associations for two categorical variables tested in a contingency table for small sample sizes; values <0.05 were considered statistically significant.

## Data Availability

Unprocessed WGS data and accompanying metadata for the *M. chimaera* (*n* = 93) and *M. abscessus* (*n* = 86) isolates sequenced in this study were deposited in the NCBI Sequence Read Archive (SRA) (Table S4) and BioProject PRJNA1458261. Additionally, we have incorporated data downloaded from SRA from two previous publications (18, 20) for global comparisons.

## SUPPLEMENTAL MATERIAL

Supplemental figures aem.00269-26-s0001.pdf Fig. S1 to S5.

Supplemental methods aem.00269-26-s0002.docx Environmental sample collection and DNA extraction and identification methods; DNA extraction and identification.

Table S1 aem.00269-26-s0003.xlsx All sample metadata.

Table S2 aem.00269-26-s0004.xlsx ENV NTM species by rpoB.

Table S3 aem.00269-26-s0005.xlsx Environmental samples from which more than one species were recovered and identified.

Table S4 aem.00269-26-s0006.xlsx SRA accession numbers.

ASM does not own the copyrights to Supplemental Material that may be linked to, or accessed through, an article. The authors have granted ASM a non-exclusive, world-wide license to publish the Supplemental Material files. Please contact the corresponding author directly for reuse.
