# Supplementary material for: Novel Hawai’i and Pacific Island circulating clusters of Mycobacterium intracellulare subsp. chimaera
Source: Appl Environ Microbiol. 2026 Jun 10;92(7):e00269-26. doi: 10.1128/aem.00269-26 (PMC13390491; doi:10.1128/aem.00269-26)
Supplement: Supplemental figures — Fig. S1 to S5. [file aem.00269-26-s0001.pdf]

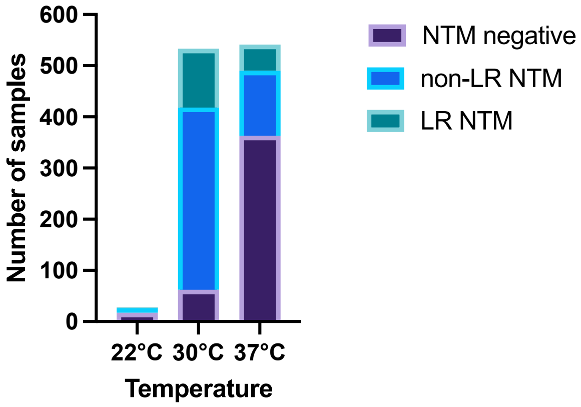

Figure S1: Comparison of NTM culture positivity across incubation temperatures. Culture positivity across the two primary incubation temperatures (30°C and 37°C) and the 22° temperature which was only used to incubate water filters from filtered freshwater stream samples. Culture results are divided into three groups: NTM culture negative (the sample was incubated at the temperature and NTM was not recovered), NTM culture positive at the incubation temperature but LR NTM was not identified, and the sample was NTM culture positive at the incubation temperature from which LR NTM was recovered and identified.

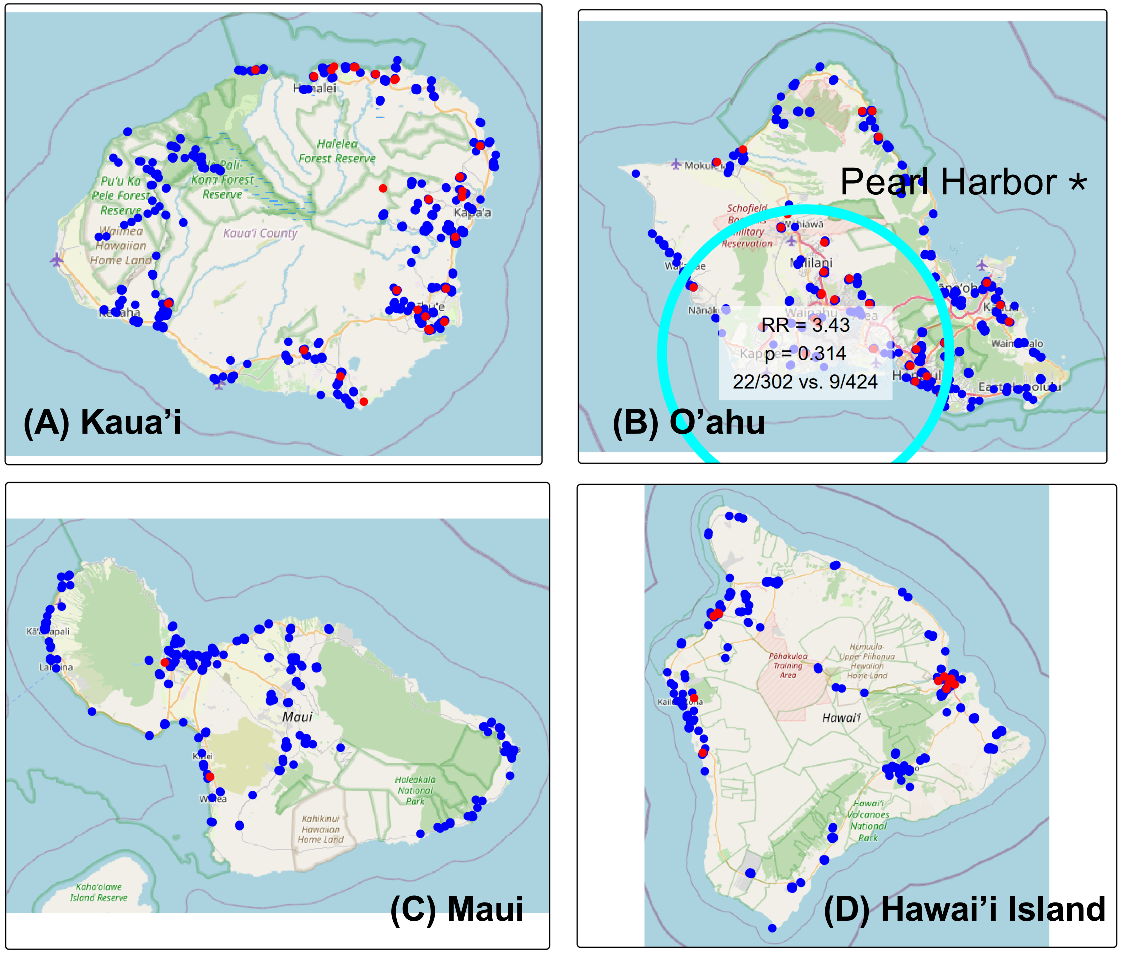

Figure S2. Hawai'i hot spots per island for environmental *M. abscessus* recovery. Red and blue points indicate *M. abscessus* culture positive and *M. abscessus* culture negative locations, respectively from **(A)** Kaua'i, **(B)** O'ahu, **(C)** Maui, and **(D)** Hawai'i Island. Larger cyan circles highlight areas of higher relative risk (Rel. Risk) for *M. abscessus*. Rel. Risk spots of $p<0.05$ indicate a statistically significant hotspot *e.g.,* O'ahu.

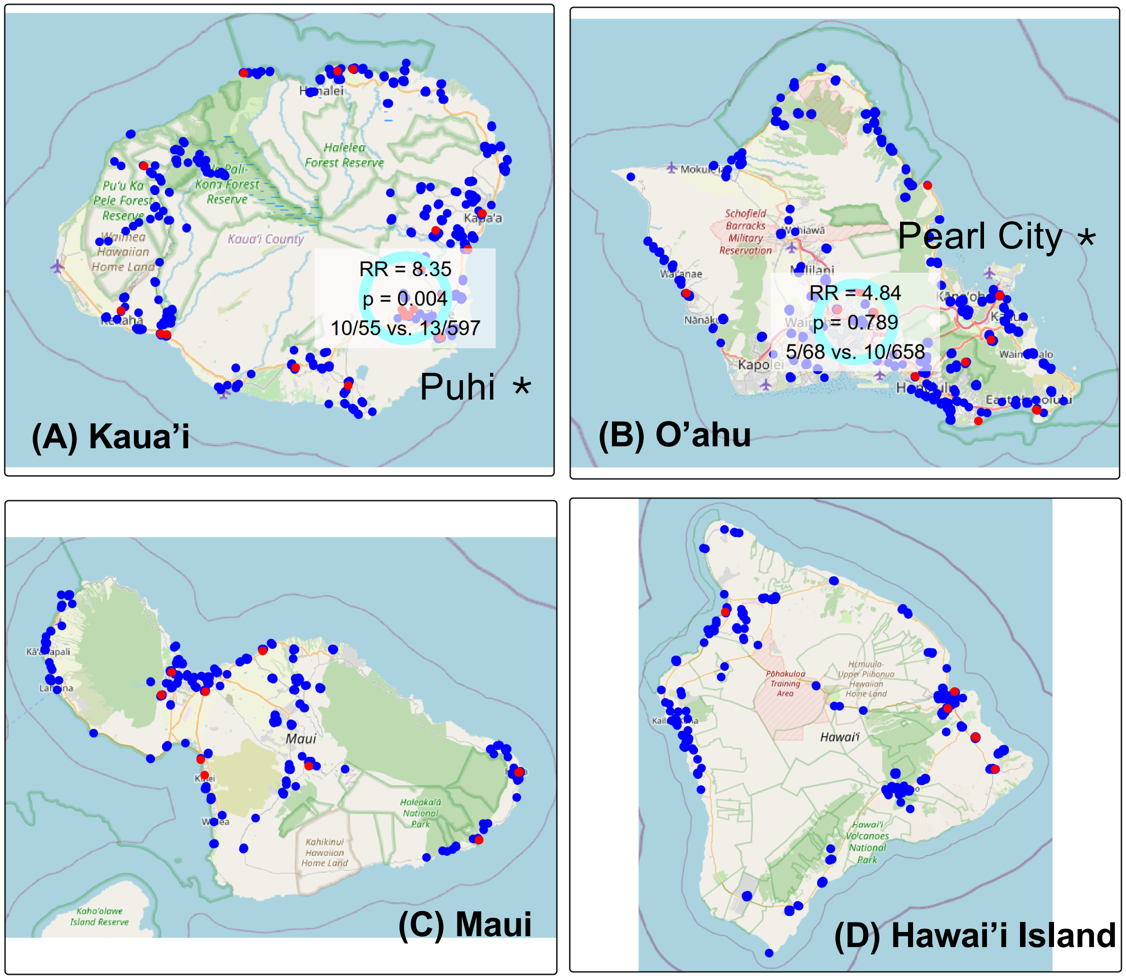

Figure S3. Hawai'i hot spots per island for environmental *M. chimaera* recovery. Red and blue points indicate *M. chimaera* culture positive and *M. chimaera* culture negative locations, respectively from (A) Kaua'i, (B) O'ahu, (C) Maui, and (D) Hawai'i Island. Larger cyan circles highlight areas of higher relative risk (Rel. Risk) for environmental NTM. Rel. Risk spots of p<0.05 indicate a statistically significant hotspot *e.g.,* Kaua'i.

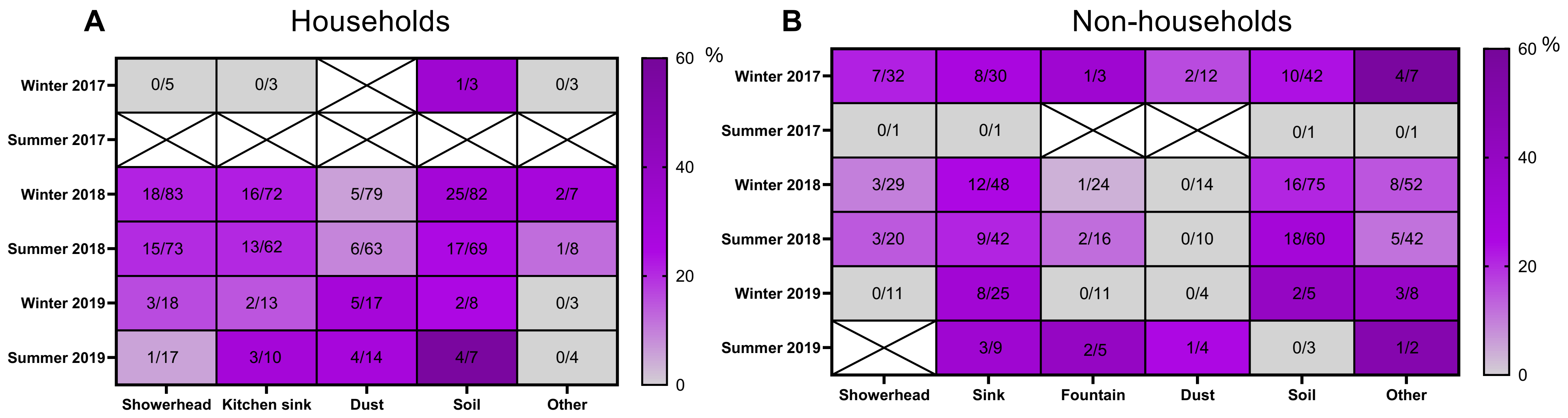

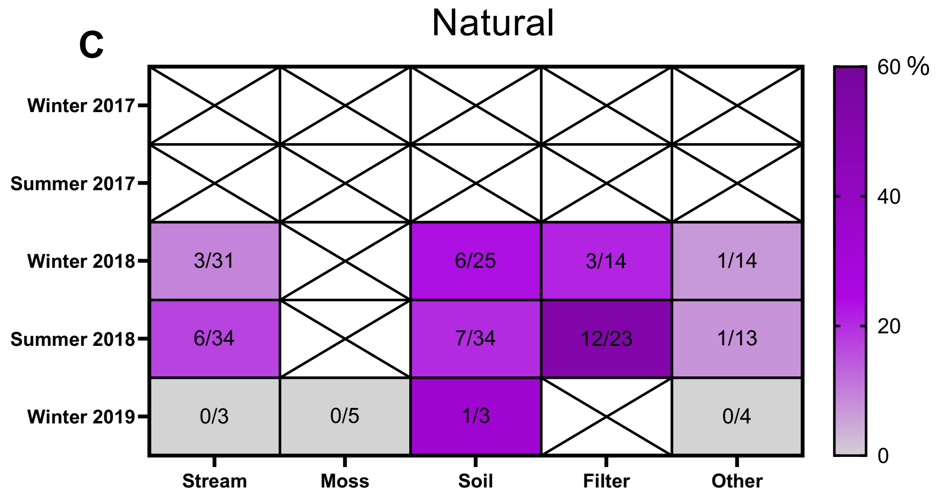

Figure S4. Non-LD NTM species are more consistently recovered over time and sample type than LR NTM. The percentage of sample types from unique households (A), non-household locations (B), and natural sources (C) that recovered NTM species that were not RR are shown as a gradient from 0% (grey) to 60% (dark purple). White crossed boxes indicate no samples were collected for that sampling time

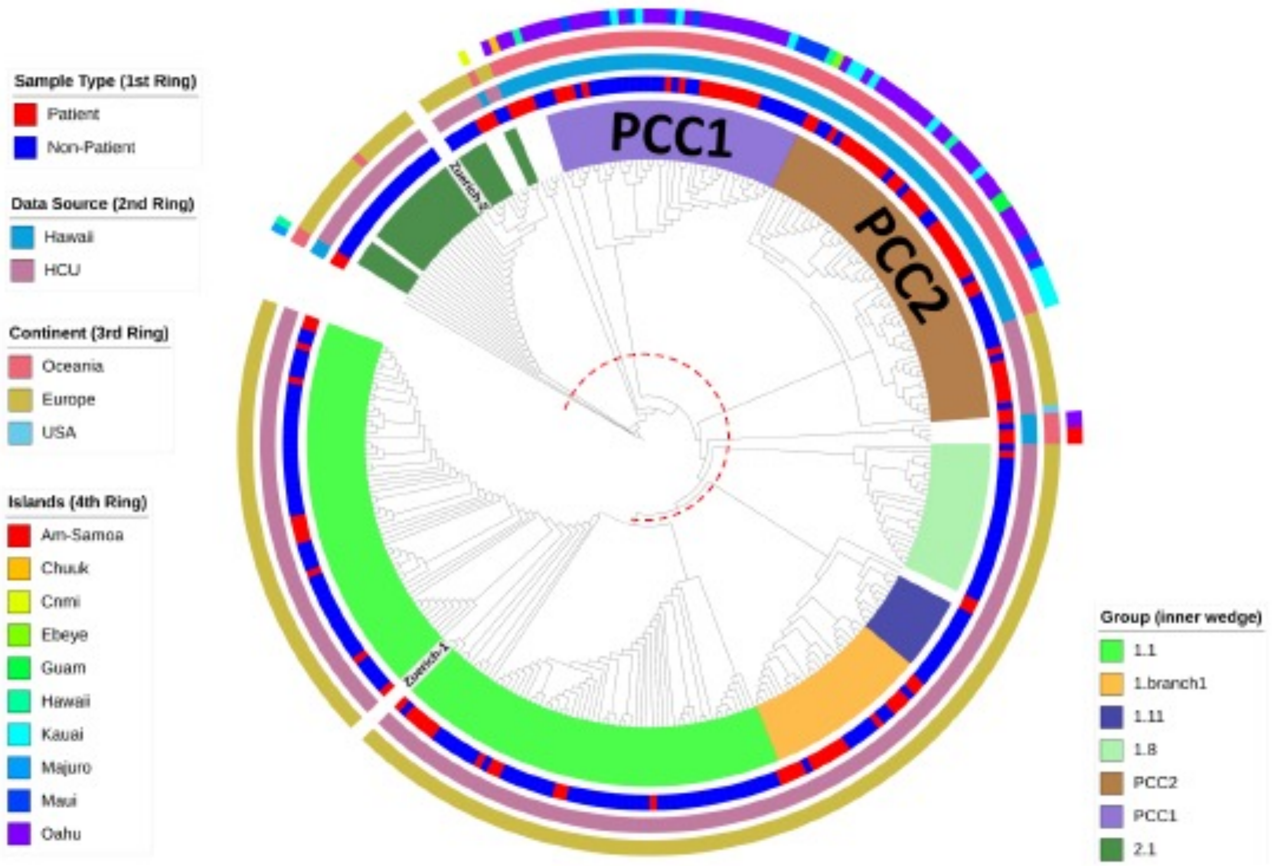

Figure S5. Cladogram shows group classifications for integrated *M. chimaera* dataset from an HCU outbreak study (n=223) and Hawai'i/Pacific Island *M. chimaera* isolates (n=92). Leaves are color-coded according to the phylogenetics groups from the previously defined HCU outbreak study and the newly identified Pacific Island Circulating Cluster 1 and 2 (PCC1 and PCC2). Annotations in the 1st ring indicated patient or non-patient sources of isolates. Annotations in the 2nd ring indicated the names of project. Annotations in the 3rd and 4th rings indicated geographic locations of sample collections. Red dash line indicates the position of branch height cut to identify major clades.
