# Supplementary material for: Novel Hawai’i and Pacific Island circulating clusters of Mycobacterium intracellulare subsp. chimaera
Source: Appl Environ Microbiol. 2026 Jun 10;92(7):e00269-26. doi: 10.1128/aem.00269-26 (PMC13390491; doi:10.1128/aem.00269-26)
Supplement: Supplemental methods — Environmental sample collection and DNA extraction and identification methods; DNA extraction and identification. [file aem.00269-26-s0002.docx]

**Supplemental Methods**

**Novel Pacific Island circulating clusters of *Mycobacterium intracellulare* subsp. *chimaera* from Hawai’i**

Rachel N. Wilsey^1ⴕ^, Liang-Hao Ding^1ⴕ^, Stephanie N. Dawrs^2,$, ⴕ^, Chelsea K. Raulerson^1^, Grant J. Norton^2,‡^, Ravleen Virdi^2,^^, Nabeeh A. Hasan^2,§^, L. Elaine Epperson^2^_,_ Stephen T. Nelson^3^, Brady Holst^4^, Yvonne L. Chan^5^, Kara Kitamura^6^, Jonathan D. Awaya^7^, Sally V. Irwin^8^, Steven Cornell^9^, Mark B. Cannon^10^, Sara Anglin^11^, Marisa K. Chelius^12,&^, Robert Hutchinson^13^, Sarah Kern^14,**^, Abi Stearns^15,Ψ^, Michael Strong^2^, Edward D. Chan^16,17,18^, James L. Crooks^4^ and Jennifer R. Honda^1,*^

**Supplementary Methods**

**Environmental sample collection and DNA extraction and identification**

**methods**

*Water biofilm samples*

We collected freshwater biofilm samples using sterile synthetic dual-tipped swabs (Puritan HydraFlock Sterile Flocked Collection Devices #25-3306 2HBT, Guilford ME) on a variety of natural surfaces previously shown to harbor NTM (Table 1) (1). We immersed and vortexed swabs in 2 mL of autoclaved ultrapure water in 5ml sterile screw cap tubes and transferred 450 µL of each sample to sterile tubes with 50 µL of 1% cetylpyridinium chloride (CPC). After vortexing a second time, we incubated samples at room temperature for 30 minutes before vortexing a third time and spreading 100 µL in duplicate onto Middlebrook 7H10 agar plates supplemented with 0.5%

glycerol and 10% oleic acid albumin enrichment (2). We incubated one plate at 30°C and the other at 37°C for 21 days then examined for colony growth.

*Water filter samples*

We collected water samples by hand-filtering 0.5-1 L of water through 0.2µm syringe filters at the stream source. After collection, filters were stored at 4°C for 1-30 days before shipment. We then extracted filter membranes from filter cartridges using snip shears sterilized with 70% ethanol. After cutting each filter membrane into four pieces using sterile forceps and razor blades, we transferred them into 5ml screw cap tubes containing two ml of autoclaved ultrapure water. After vortexing on high for 30 seconds, we transferred 450 µL of each sample to sterile tubes with 50 µL of 1% CPC, vortexed again and incubated at room temperature for 30 minutes. We vortexed samples a third time before spreading 100 µL in triplicate on 7H10 agar plates supplemented with oleic acid/glycerol enrichment. We incubated plates at 22°C, 30°C and 37°C for 21 days then examined them for colony growth.

*Dust*

Dust samples were collected from household and non-household sources with a visible accumulation of dust using sterile synthetic dual-tipped swabs (Puritan HydraFlock Sterile Flocked Collection Devices #25-3306 2HBT, Guilford ME). We immersed the dust swabs in 2 ml of an enzymatic solution containing 1 mg/ml pronase, 1 mg/ml lipase, 1 mg/ml cellulase, 1% Tween 80, and 1% NaCl. Samples were then vortexed briefly and incubated on a rocking platform at 37°C for 1 hour. 450 µL was then transferred to sterile tubes with 50 µL of 1% CPC, vortexed, and incubated at room temperature for 30 minutes, at which point 100 µL was spread in duplicate onto 7H10 agar plates supplemented with glycerol, oleic acid albumin enrichment, and amphotericin B. One plate was incubated at 30°C and the other at 37°C for 21 days, at which point colony growth was determined.

*Soil*

We collected soil by clearing away surface debris to expose top soil, then scooping soil into sterile 50ml conical vials. We immersed 1g of soil into 10 ml of enzymatic solution containing 1mg/ml pronase, 1mg/ml lipase, 1mg/ml cellulase, 1% Tween 80 and 1% NaCl (3). After briefly vortexing on high, we incubated samples on a rocking platform at 37°C for 1 hour then allowed them to settle upright for 1 hour. Once soil particles had settled, we transferred 450 µL to sterile tubes with 50 µL of 1% CPC, vortexed again and incubated at room temperature for 30 minutes. Following one more vortex, we spread 100 µL in duplicate on 7H10 agar plates supplemented with oleic acid/glycerol enrichment and amphotericin B. We incubated one plate at 30°C and the other at 37°C for 21 days then examined them for colony growth.

**DNA Extraction and identification**

We picked and inoculated grown colonies into Middlebrook 7H9 broth to create bacterial stocks then centrifuged 1 mL of turbid bacterial stock at 13,000 xg for 1 min to create bacterial pellets. We stored bacterial pellets at -80°C until used for DNA isolation, following the DNA extraction and species identification procedures by *rpoB* gene sequencing. Bacterial pellets were thawed from frozen and followed established methods for DNA extraction (4). Specifically, we immersed bacterial pellets in 100 µL of lysis buffer and 25 µL of 100mg/mL lysozyme, vortexed briefly then incubated overnight at 37°C. We added 25 µL of 2.5mg/mL proteinase k and 50 µL of 20% SDS, then incubated for 1 hour at 37°C. We transferred the entire volume of each sample to a deep well 96-well plate containing 100 µL of zirconia beads and added 400 µL of ChIP binding buffer from the ZR-96 Genomic DNA Clean and Concentrator-5 kit (Zymo Research, Cat No. D4067) to each well. Using a Qiagen TissueLyzer II, we bead beat the deep well plate for 3 minutes at 30 Hz then centrifuged at maximum speed for 2 minutes to pellet debris. We transferred the sample supernatants over to the column provided with the kit and the remainder of the DNA extraction followed the manufacturer’s instructions.

To determine the identity of the cultured environmental NTM, we performed Sanger sequencing of a ~700 bp region of the RNA polymerase Region Five beta subunit (*rpoB*) gene. We set up and submitted PCR reactions for Sanger sequencing (Quintara Biosciences, San Francisco, CA, USA) which included 1-10 ng of DNA template, 4 µL mixture of 5 µM forward and reverse primers, and nuclease free water. We trimmed the resulting sequences for quality and compared them to sequences in the National Center for Biotechnology Information (NCBI) Genbank using the BLAST algorithm. Isolates were identified to the species level with call criteria of >90% query coverage, >90% ANI, and >630 bp sequence length and the best match determined by E-value. Putatively “novel” NTM were defined as isolates that highly matched (greater than 90% BLAST identity and coverage) to uncharacterized mycobacteria in the NCBI BLAST GenBank database.

References

1. Honda JR, Virdi R, Chan ED. 2018. Global Environmental Nontuberculous Mycobacteria and Their Contemporaneous Man-Made and Natural Niches. Front Microbiol 9:2029.

2. Virdi R, Lowe ME, Norton GJ, Dawrs SN, Hasan NA, Epperson LE, Glickman CM, Chan ED, Strong M, Crooks JL, Honda JR. 2021. Lower Recovery of Nontuberculous Mycobacteria from Outdoor Hawai'i Environmental Water Biofilms Compared to Indoor Samples. Microorganisms 9.

3. Thorel MF, Falkinham JO, 3rd, Moreau RG. 2004. Environmental mycobacteria from alpine and subalpine habitats. FEMS Microbiol Ecol 49:343-7.

4. Epperson LE, Strong M. 2020. A scalable, efficient, and safe method to prepare high quality DNA from mycobacteria and other challenging cells. J Clin Tuberc Other Mycobact Dis 19:100150.
